# Partial genome sequence of *Thioalkalivibrio thiocyanodenitrificans* ARhD 1^T^, a chemolithoautotrophic haloalkaliphilic sulfur-oxidizing bacterium capable of complete denitrification

**DOI:** 10.1186/s40793-015-0080-3

**Published:** 2015-10-26

**Authors:** Tom Berben, Dimitry Y. Sorokin, Natalia Ivanova, Amrita Pati, Nikos Kyrpides, Lynne A. Goodwin, Tanja Woyke, Gerard Muyzer

**Affiliations:** Microbial Systems Ecology, Department of Aquatic Microbiology, Institute of Biodiversity and Ecosystem Dynamics, University of Amsterdam, Amsterdam, The Netherlands; Winogradsky Institute of Microbiology, RAS, Moscow, Russia; Department of Biotechnology, Delft University of Technology, Delft, The Netherlands; Joint Genome Institute, Walnut Creek, CA USA

## Abstract

*Thioalkalivibrio thiocyanodenitrificans* strain ARhD 1^T^ is a motile, Gram-negative bacterium isolated from soda lakes that belongs to the *Gammaproteobacteria*. It derives energy for growth and carbon fixation from the oxidation of sulfur compounds, most notably thiocyanate, and so is a chemolithoautotroph. It is capable of complete denitrification under anaerobic conditions. The draft genome sequence consists of 3,746,647 bp in 3 scaffolds, containing 3558 protein-coding and 121 RNA genes. *T. thiocyanodenitrificans* ARhD 1^T^ was sequenced as part of the DOE Joint Genome Institute Community Science Program.

## Introduction

Soda lakes are formed in inland arid areas where ground water, rich in CO_2_/bicarbonate, but poor in divalent cations (calcium and magnesium), accumulates in basins and evaporates. The resulting system has a stable high pH above 9 and up to 11, high soluble carbonate alkalinity reaching molar concentrations and moderate to extremely high salinity [1]. Despite these extreme characteristics, a rich microbial community is found to thrive in such lakes, driving highly active biogeochemical cycles. Thus far, knowledge on the dynamics of and the connections between these cycles is limited [2]. A better understanding of the biogeochemistry and the microbial species involved will lead to clearer insights into the ecology of soda lakes. Our research focuses on the species involved in the sulfur cycling in hypersaline soda lakes. To learn more about the community involved in the oxidizing part of the cycle, we have sequenced a large number of strains of the dominant cultivated haloalkaliphilic sulfur-oxidizing bacteria belonging to the genus *Thioalkalivibrio*. Here we present the partial genome sequence of *Thioalkalivibrio thiocyanodenitrificans* ARhD 1^T^.

## Organism information

### Classification and features

**Fig. 1:**
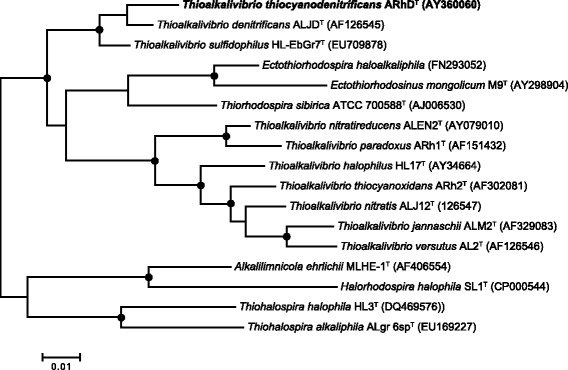
16S rRNA phylogenetic tree of the described *Thioalkalivibrio* species, as well as various organisms belonging to the family of *Ectothiorhodospiraceae*. Nodes with a bootstrap value between 90-100 % are marked with black dots. The outgroup, members of the *Alphaproteobacteria*, are pruned from the tree. The tree was constructed in ARB [15] and the bootstrap values calculated using MEGA6 [16]

**Fig. 2:**
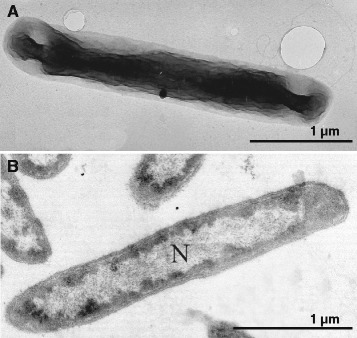
Electron microscopy photographs of strain ARhD1 grown with thiocyanate. (**a**) cell with a polar flagellum positively stained by uranyl acetate; (**b**) thin section showing Gram-negative cell ultrastructure and extended nucleoid (N)

*T. thiocyanodenitrificans* ARhD 1^T^ is a Gram-negative bacterium belonging to the *Gammaproteobacteria* (Fig. 1). It is a motile rod with dimensions 0.4–0.6 × 1.5–5 μm (Fig. 2). Basic information about the organism is summarized in Table 1. It is obligately chemolithoautotrophic and haloalkaliphilic. Energy is derived from the oxidation of a variety of inorganic sulfur compounds including sulfide, thiosulfate, thiocyanate, polysulfide, elemental sulfur and tetrathionate. It is facultatively anaerobic, capable of growth with nitrate or nitrite as electron acceptor when thiosulfate or thiocyanate serves as electron donor, although anaerobic growth with thiocyanate is extremely slow (0.006 h^−1^ compared to 0.032 h^−1^ in the presence of oxygen). At present, *T. thiocyanodenitrificans* is the only sulfur-oxidizing bacterium for which anaerobic growth with thiocyanate has been proven. The final product of nitrite reduction is N_2_. Since nitrite cannot be assimilated, *T. thiocyanodenitrificans* can only use either external ammonia or ammonia derived from thiocyanate as a nitrogen source [3].

**Table 1:** Classification and general features of *Thioalkalivibrio thiocyanodenitrificans* ARhD 1^T^ [17]

MIGS ID	Property	Term	Evidence code^a^
	Classification	Domain *Bacteria*	TAS [18]
		Phylum *Proteobacteria*	TAS [19, 20]
		Class *Gammaproteobacteria*	TAS [20, 21]
		Order *Chromatiales*	TAS [20, 22]
		Family *Ectothiorhodospiraceae*	TAS [23]
		Genus *Thioalkalivibrio*	TAS [24]
		Species *Thioalkalivibrio thiocyanodenitrificans*	TAS [3, 25]
		Type strain: ARhD 1^T^ (DSM 16954)	
	Gram stain	Negative	TAS [3, 24]
	Cell shape	Rod	TAS [3]
	Motility	Motile	TAS [3]
	Sporulation	Non-sporulating	NAS
	Temperature range	Mesophilic	TAS [3]
	Optimum temperature	33–35 °C	TAS [3]
	pH range; Optimum	8.0–10.3	TAS [3]
	Carbon source	Inorganic carbon	TAS [3]
MIGS-6	Habitat	Soda lakes	TAS [3]
MIGS-6.3	Salinity	0.3–2 M Na^+^	TAS [3]
MIGS-22	Oxygen requirement	Facultative anaerobe	TAS [3]
MIGS-15	Biotic relationship	Free-living	NAS
MIGS-14	Pathogenicity	Non-pathogenic	NAS
MIGS-4	Geographic location	Wadi Natrun, Egypt	TAS [3]
MIGS-5	Sample collection	2002	TAS [3]
MIGS-4.1	Latitude	Not reported	
MIGS-4.2	Longitude	Not reported	
MIGS-4.4	Altitude	Not reported	

^a^Evidence codes - IDA: Inferred from Direct Assay; TAS: Traceable Author Statement (i.e., a direct report exists in the literature); NAS: Non-traceable Author Statement (i.e., not directly observed for the living, isolated sample, but based on a generally accepted property for the species, or anecdotal evidence). These evidence codes are from the Gene Ontology project [26]

## Genome sequencing information

### Genome project history

This genome sequence is part of a large project aimed at sequencing approximately 70 *Thioalkalivibrio* isolates. *T. thiocyanodenitrificans* ARhD 1^T^ was specifically selected for its ability to grow on thiocyanate as its sole electron donor, both in the presence and absence of oxygen. This is interesting not only in terms of microbial physiology, but also in biotechnology, where thiocyanate is a waste product in mining effluents [4]. The permanent draft genome presented here contains approximately 3.7 million basepairs in 3 scaffolds. It was sequenced at the Joint Genome Institute as part of project 401911 and released in August 2012. A summary of important information regarding the sequencing project is shown in Table 2.

**Table 2:** Project information

MIGS ID	Property	Term
MIGS 31	Finishing quality	Improved high-quality draft
MIGS-28	Libraries used	Illumina short and long insert paired-end
MIGS 29	Sequencing platforms	Illumina HiSeq 2000
MIGS 31.2	Fold coverage	2322
MIGS 30	Assemblers	ALLPATHS R39750 [7], Velvet 1.1.05 [8], PHRAP 4.24
MIGS 32	Gene calling method	Prodigal [12], GenePRIMP [13]
	Locus Tag	THITHI
	Genbank ID	AQZO00000000
	GenBank Date of Release	2012-08-13
	GOLD ID	Ga0025308
	BIOPROJECT	PRJNA81091
	IMG submission ID	10076
MIGS 13	Source Material Identifier	DSM 16954
	Project relevance	Biotechnology

## Growth conditions and genomic DNA preparation

*T. thiocyanodenitrificans* ARhD 1^T^ (DSM 16954) was grown under aerobic conditions in a standard sodium carbonate-bicarbonate buffer at pH 10 and 0.6 M Na^+^ with 40 mM thiosulfate as an energy source [5]. The cells were stored at −80 °C after harvesting by centrifugation. Genomic DNA was extracted using a phenol-chloroform-isoamylalcohol approach. The cell pellet was suspended in Tris-EDTA (pH 8) and lysed using SDS and proteinase K. DNA was extracted using the phenol-chloroform-isoamylalcohol mixture and precipitated with ethanol. The resulting pellet was dried and dissolved in water. Extraction yield and quality were measured using the DNA Mass Standard Kit provided by the JGI.

## Genome sequencing and assembly

The draft genome of *Thioalkalivibrio thiocyanodenitrificans* ARhD 1^T^ was generated at the DOE Joint Genome Institute (JGI) using Illumina sequencing [6]. For this genome, we constructed and sequenced an Illumina short-insert paired-end library with an average insert size of 270 bp which generated 41,681,874 reads and an Illumina long-insert paired-end library with an average insert size of 8291 +/− 2700 bp which generated 18,699,268 reads totaling 9,057 Mbp of Illumina data. All general aspects of library construction and sequencing performed are available at the JGI web site. The initial draft assembly contained 42 contigs in 12 scaffold(s) and was assembled with ALLPATHS, version 39,750 [7], and the consensus was computationally shredded into 10 kbp overlapping fake reads (shreds). The Illumina draft data was also assembled with Velvet, version 1.1.05 [8], and the consensus sequences were computationally shredded into 1.5 Kbp overlapping fake reads (shreds). The Illumina draft data was assembled again with Velvet using the shreds from the first Velvet assembly to guide the next assembly. The consensus from the second Velvet assembly was shredded into 1.5 Kbp overlapping fake reads. The fake reads from the ALLPATHS assembly and both Velvet assemblies and a subset of the Illumina CLIP paired-end reads were assembled using parallel phrap, version 4.24 (High Performance Software, LLC). Possible mis-assemblies were corrected with manual editing in Consed [9–11]. Gap closure was accomplished using repeat resolution software (Wei Gu, unpublished), and sequencing of bridging PCR fragments with Sanger and/or PacBio (unpublished, Cliff Han) technologies. A total of 18 PCR PacBio consensus sequences were completed to close gaps and to raise the quality of the final sequence. The total estimated size of the genome is 3.7 Mb and the final assembly is based on 9,057 Mbp of Illumina draft data, which provides an average 2,322X coverage of the genome. The Genbank record for this genome contains three annotated scaffolds (accessions NZ_KB900536-8) and eight, redundant, unannotated (accessions AQZO01000001-8) scaffolds. The eight unannotated scaffolds have been merged into three, which were subsequently annotated and described in this report.

## Genome annotation

Genes were predicted using Prodigal [12], followed by pseudogene detection using GenePRIMP [13]. The predicted genes were translated and annotated using the NCBI’s NR database in combination with the UniProt, TIGRFam, Pfam, KEGG, COG and InterPro databases and tRNAScanSE [14] for tRNA prediction. Ribosomal RNAs were detected using models built from SILVA. Further annotation was performed using the Integrated Microbial Genomes (IMG) platform. The annotation is publicly available within IMG, using submission ID 10076.

## Genome properties

The high-quality draft sequence comprises 3,746,647 bp divided in 3 scaffolds with a total GC-content of 64.8 %. Gene prediction yields 3558 protein-coding genes and 121 RNA-coding genes (Table 3). A total of 66.2 % of the protein coding genes could be assigned to COGs, with 79 % of these assigned to functional categories (Table 4).

**Table 3:** Genome statistics

Attribute	Value	% of Total
Genome size (bp)	3,746,647	100.00
DNA scaffolds	3	100.00
DNA G + C (bp)	2,428,970	64.83
DNA coding (bp)	3,274,863	87.41
Total genes	3679	100.00
Protein coding genes	3558	96.71
RNA genes	121	3.29
Pseudo genes	50	1.36
Genes in internal clusters	Not determined	Not determined
Genes with function prediction	2736	74.37
Genes assigned to COGs	2328	63.28
Genes with Pfam domains	1976	69.33
Genes with signal peptides	314	8.53
Genes with transmembrane helices	900	24.46
CRISPR repeats	3	100.00

**Table 4:** Number of genes associated with the 25 general COG functional categories

Code	Value	% age	Description
J	158	6.17	Translation, ribosomal structure and biogenesis
A	2	0.08	RNA processing and modification
K	126	4.92	Transcription
L	164	6.41	Replication, recombination and repair
B	1	0.04	Chromatin structure and dynamics
D	30	1.17	Cell cycle control, Cell division, chromosome partitioning
V	32	1.25	Defense mechanisms
T	120	4.69	Signal transduction mechanisms
M	181	7.07	Cell wall/membrane biogenesis
N	49	1.91	Cell motility
U	100	3.91	Intracellular trafficking and secretion
O	145	5.66	Posttranslational modification, protein turnover, chaperones
C	206	8.05	Energy production and conversion
G	110	4.30	Carbohydrate transport and metabolism
E	167	6.52	Amino acid transport and metabolism
F	63	2.46	Nucleotide transport and metabolism
H	119	4.65	Coenzyme transport and metabolism
I	65	2.54	Lipid transport and metabolism
P	150	5.86	Inorganic ion transport and metabolism
Q	38	1.48	Secondary metabolites biosynthesis, transport and catabolism
R	282	11.02	General function prediction only
S	252	9.84	Function unknown
-	1351	36.72	Not in COGs

The total is based on the total number of protein coding genes in the genome

## Conclusions

This genome sequence of *Thioalkalivibrio thiocyanodenitrificans* provides valuable insight into the carbon and nitrogen metabolism, and into the genes that are involved in energy conservation. Furthermore, we hope to understand the mechanism by which this organism adapts to the extreme conditions present in soda lakes. Finally, insight in the genome sequence might be helpful in improving the biotechnological application of this organism in the removal of sulfur compounds from waste streams and the bioremediation of cyanide-containing mining tailings.
